# Higher Consumption of Fruit and Vegetables Is Associated With Lower Worries, Tension and Lack of Joy Across the Lifespan

**DOI:** 10.3389/fnut.2022.837066

**Published:** 2022-05-02

**Authors:** Simone Radavelli-Bagatini, Marc Sim, Lauren C. Blekkenhorst, Nicola P. Bondonno, Catherine P. Bondonno, Richard Woodman, Joanne M. Dickson, Craig Harms, Dianna J. Magliano, Jonathan E. Shaw, Robin M. Daly, Jonathan M. Hodgson, Joshua R. Lewis

**Affiliations:** ^1^Nutrition & Health Innovation Research Institute, School of Medical and Health Sciences, Edith Cowan University, Perth, WA, Australia; ^2^Medical School, The University of Western Australia, Perth, WA, Australia; ^3^Danish Cancer Society Research Centre (DCRC), Copenhagen, Denmark; ^4^Flinders Centre for Epidemiology and Biostatistics, Flinders University, Adelaide, SA, Australia; ^5^School of Arts and Humanities (Psychology), Edith Cowan University, Perth, WA, Australia; ^6^School of Arts and Humanities, Psychology and Criminology, Edith Cowan University, Perth, WA, Australia; ^7^Diabetes and Population Health, Baker Heart and Diabetes Institute, Melbourne, VIC, Australia; ^8^Clinical Diabetes and Epidemiology, Baker Heart and Diabetes Institute, Melbourne, VIC, Australia; ^9^School of Public Health and Preventive Medicine, Monash University, Melbourne, VIC, Australia; ^10^School of Exercise and Nutrition Science, Institute for Physical Activity and Nutrition, Deakin University, Geelong, VIC, Australia; ^11^Centre for Kidney Research, School of Public Health, Sydney Medical School, Children's Hospital at Westmead, The University of Sydney, Sydney, NSW, Australia

**Keywords:** AusDiab, fruit and vegetable consumption, worries, tension, joy, stress

## Abstract

**Background and Aims:**

Higher total fruit and vegetable (FV) intakes have been associated with lower perceived stress. However, the relationship of FV intake with domains of perceived stress is unclear. The aim of this cross-sectional study was to explore the relationship between consumption of FV and four perceived stress domains (worries, tension, lack of joy and demands) in a population-based cohort of Australian adults.

**Methods:**

Participants (n = 8,640) were men and women aged ≥25 years from the Australian Diabetes, Obesity and Lifestyle (AusDiab) Study. Dietary intake was assessed using a 74-item validated Food Frequency Questionnaire. Perceived stress domains were determined using a validated 20-item version of the Perceived Stress Questionnaire, with higher scores representing higher perceived stress. Cut-offs for high perceived stress domains were obtained from the highest quartiles of each domain for each sex. Multivariable-adjusted logistic regression was performed to investigate cross-sectional associations.

**Results:**

The mean age of participants (50.1% females) was 47.8 (SD 15) years. Those with higher intakes of FV, combined and separately, had a significantly lower odds (16–36%) for higher worries, tension and lack of joy, independent of other lifestyle factors.

**Conclusion:**

In Australian adults, higher consumption of FV was associated with lower odds of worries, tension and lack of joy. Following the dietary guidelines for the recommended intake of FV may help improve feelings of worries, tension and lack of joy, which are linked to mental health problems.

## Introduction

Prolonged exposure to stress can be a risk factor for chronic illnesses (1), having been linked to mental health issues such as anxiety and depression (2, 3), as well as physical health issues (4), including cardiovascular disease (CVD) (5, 6). The number of people affected by stress as well as mental health issues continue to grow globally, with depression reported to affect more than 300 million people worldwide (7).

Increasing evidence shows that fruit and vegetable-rich diets may have a protective role on stress, with consumption of whole fruit and vegetables (8), as well as some specific types (such as citrus fruits and leafy green vegetables) being linked with less stress (9). Moreover, healthy lifestyle factors, such as being active and adhering to a healthy diet (10, 11), particularly a fruit and vegetable-rich diet, can help alleviate mental health issues (10, 12–14) and improve mental well-being (15). Therefore, greater intakes of FV could help reduce the estimated 7.8 million premature deaths worldwide, which have been associated with low intakes of fruit and vegetables (16), and consequently decrease the additional burden of chronic diseases and deaths caused by mental health worldwide (7).

It is noteworthy that this relationship is likely to be bidirectional, as stress can also lead to overeating (17, 18) and poor dietary choices, such as increased intake of sugar and fat-rich foods (19, 20). Growing evidence suggests that adherence to a healthy diet (e.g., rich in FV and low in sugar and fat) may improve well-being (21). Bioactive compounds found in FV, such as vitamins (e.g., B and C vitamins), minerals (e.g., zinc, magnesium) and fiber seem to modulate neurochemicals (e.g., endorphin, dopamine, serotonin, and norepinephrine) in the brain that promote feelings of happiness (22). Similarly, polyphenols and carotenoids found in FV can reduce oxidative stress (23) and inflammation (24) that have also been linked with improved well-being. Thus, studies investigating the relationship between diet and stress may contribute to a healthier lifestyle and help reduce stress-related conditions. Exploring the association of FV intake with specific stress domains (25), such as “worries,” “tension,” “joy,” and “demands” (stressor) (25) will allow for more precise understanding of this relationship. By definition, “worry” is “a feeling of being unhappy and frightened about something” (26), it is the repetitive feeling of potential future harmful life events (27). This domain covers “worries, anxious concern for the future, and feelings of desperation and frustration” (25). “Tension” is “a feeling of nervousness before an important or difficult event” (26). This domain explores “tense disquietude, exhaustion and the lack of relaxation” (25). “Joy” is defined as a “feeling of great pleasure and happiness” (26). This domain includes “positive feelings of challenges, joy, energy, and security” (25). “Demands” is defined as “the difficult things that you have to do” (26). This domain covers “perceived environmental demands, such as lack of time, pressure, and overload,” and it is considered an environmental stressor, rather than a stress reaction (25).

Therefore, the aim of this study was to examine the relationship between consumption of FV and four perceived stress domains (worries, tension, joy, and demands) in a population-based cohort of Australian adults.

## Materials and Methods

### Study Population

This study includes participants from the Australian Diabetes, Obesity, and Lifestyle (AusDiab) study, a large prospective study which aimed to determine the prevalence of diabetes, and other cardiovascular risk factors, including obesity, hypertension, and dyslipidaemia in a cohort representative of the general Australian population. The AusDiab study collected data from 42 randomly selected census collector districts across Australia in 1999–2000, as previously reported (28). A stratified cluster sampling method was used to recruit Australian men and women from six states and the Northern Territory (29). A total of 11,247 participants aged ≥25 years attended a clinical examination (5,049 men; 6,198 women) and were enrolled in the study (28). More details have been previously reported (30).

A flow diagram for participants included in this study is shown in Supplementary Figure 1. We excluded participants with missing data for perceived stress (*n* = 1,697) and dietary intake (*n* = 148), as well as those missing data for confounding factors (*n* = 436). We also excluded participants with extremely low or high energy intake (<3,300 or >17,500 kJ in men and <2,500 kJ or >14,500 kJ in women) (*n* = 275) (31), and pregnant women (*n* = 51), as per previous studies (8, 14). After exclusions (*n* = 2,607), 8,640 were deemed eligible for the study.

### Assessment of Perceived Stress Domains

Perceived stress was assessed over the previous 12 months using the original perceived stress questionnaire (PSQ) developed by Levenstein et al. (32). Different stress domains were calculated using the perceived stress domains 20-item reduced PSQ, validated by Fliege and colleagues (25), in which the authors selected the statements that showed the highest item-scale-correlation. This resulted in four stress domains (“worries,” “tension,” “joy” [the answers were reversed, therefore referred to as “lack of joy”], and “demands”), each one containing five items (25). “Worries” comprise feelings of anxiety in relation to the future, desperation, and frustration. “Tension” includes tense agitation, exhaustion, and the lack of relaxation. “Joy” relates to positive feelings of challenge, energy, and security. Finally, “demands” covers perceived environmental demands, such as lack of time, pressure, and overload (25). All items were scored on a four-point scale ranging from one (“almost never”) to four (“usually”), with positive questions (Questions 1, 7, 10, 13, 17, 21, 25, 29), being reverse scored (four [“almost never”] to one [“usually”]). The scores for each domain were derived from five specific questions (25), as shown in Supplementary Table 1. A total score was calculated for each domain, ranging from a minimum of 5 to a maximum of 20 (25), with higher scores indicating higher perceived stress levels (33). This questionnaire showed high internal consistency (Cronbach's α = 0.85) (25). Specific cut-points for each domain were calculated based on the highest quartile of raw scores for men and women separately (Supplementary Table 2), since perceived stress is usually higher in women compared to men (34, 35). As there are no pre-defined cut-offs for the perceived stress domains, we assume the 25% in the highest quartiles presented higher perceived stress. These specific cut-point for worries, tension, lack of joy, and demands were equal to or higher than 9, 11, 11, 12 for men; and equal to or higher than 10, 12, 12, 13 for women, respectively.

### Dietary Intake

A validated food frequency questionnaire (FFQ) from the Cancer Council of Victoria (36) was used to assess dietary intake at baseline (1999–2000). This questionnaire comprises 74 food items and recalls usual food intake in the previous 12 months. Total food consumption was computed based on intakes of each food (i.e., bread, milk, FV) in grams per day, calculated according to season, to estimate total energy intake (Kj/d) (36). Nutrient intakes were assessed by using the NUTTAB95 food composition database. Alcohol intake was assessed based on alcoholic beverages frequency of consumption (varying from “never” to “every day”) (36).

### Intake of Fruit and Vegetables

Intake of fruit and vegetables were assessed using the FFQ and estimated based on the frequency of consumption in the previous 12 months, as previously reported (8). Total fruit included 11 different types (Supplementary Table 3), excluding fruit juice and tinned fruits, due to their high sugar content. The recommendations of the Australian Dietary Guidelines is to consume fresh fruit, with consumption of fruit juice, tinned and dried fruits being recommended as substitutes for fresh fruit only occasionally (37). Total vegetable intake included 24 types (Supplementary Table 3), excluding potatoes roasted and fried—including hot chips, as these are not recommended as part of a healthy diet (37). The daily intake was estimated in grams per day (g/d). Serves of fruit and vegetables were calculated by dividing the total intake in grams by the recommended serving size (37): for fruit, one serve equals 150 g/day; and for vegetables one serve equals 75 g/day (37).

### Demographic and Clinical Assessment

The following demographic variables were collected during household interviews: date of birth, sex (male/female), relationship status (married, single, de facto, separated, divorced, widowed), and education status (never to some high school, completed University or equivalent). Relationship status (38) and level of education (39) have been added as potential confounding factors in our study as they can affect stress and mental health. Furthermore, higher educational level (40) and being married (41) have also been associated with greater FV intakes. The socio-economic index for areas (SEIFA), which assesses the socio-economic status per geographic area according to the 5-yearly census (42) was used to estimate socio-economic status. SEIFA is an Index of Relative Socio-economic Disadvantage (IRSD) (43). Anthropometric measurements were performed at the clinic visits (28, 44) and included: height, measured using a stadiometer to the nearest 0.5 cm without shoes; and weight, measured without shoes and excess clothing, using a mechanical beam balance to the nearest 0.1 kg (45). Body mass index (BMI) was calculated as weight (kg) divided by height in meters squared.

Physical activity in the previous week was estimated using the Active Australia Survey Questionnaire (46). Total walking time (if continuous for ≥ 10 min) and/or moderate-intensity physical activity, were summed with the time performing vigorous-intensity activity (multiplied by two) (46) to obtain “total physical activity” (min/week). Levels of physical activity were classified as sufficient (>150 min per week across at least five sessions), insufficient (<150 min per week and/or fewer than five sessions per week), or no physical activity (46).

Smoking status was assessed using an interviewer-administered questionnaire (28), and classified as: current smokers (smoked at least daily), ex-smokers (smoked less than daily for at least the last 3 months), or never smokers (smoked <100 cigarettes during life) (47).

Plasma glucose levels was assessed and categorized as normal glucose levels, impaired fasting glucose, impaired glucose tolerance, newly diagnosed diabetes mellitus (DM), or known DM. Prior cardiovascular disease (CVD) was self-reported by answering yes/no (48). Variables indicating diabetes and CVD status were added to the multivariable adjusted model as potential confounding factors, as those participants may have received dietary advice promoting a healthier lifestyle. Average weekly income was estimated based on six categories: $0–199, $200–399, $400–599, $600–799, $800–1,499, and >$1,500.

The Dietary Guideline Index (DGI) comprised of 15 items—including fruit and vegetable intakes (49), based on the Australian Dietary Guidelines (50, 51), was used as a marker of diet quality. Items were scored from 0 to 10 (lowest to optimal intake) and total scores ranged from 0 to 150 (higher scores represent better diet quality and adherence to dietary guidelines) (49).

Life events scale comprised 13 potential life events (i.e., marriage breakdown, financial hardship, and death of a family member/friend in the 12 months (52). Participants were asked how much those life events affected them in the previous 12 months (with responses being: 0 = no event, 1 = had minimum or no effect, 2 = affected a little, 3 = affected somewhat, and 4 = affected a great deal). Participants were given a score of “0” for the items they selected “no life event”; or a score of “1” to each item their response was ≥1. Items were summed with scores ranging from 0 to 13.

A physical component score (PCS) assessed using the SF-36 (36-item short form survey health-related quality of life scale) (53) was added to the multivariable adjusted model, to control for physical issues. SF36 is composed of 8 domains comprising two summary measures: physical (four domains: physical-functioning, role-physical, bodily pain, and general health) and mental health (four domains: vitality, social functioning, role-emotional, and mental health). In the present study, only the physical health score was used (since the mental health measure would have been an overadjustment). Internal consistency (Cronbach's alpha) for the eight domains was 0.83–0.93, 0.94 for the physical health summary score, and 0.89 for the mental health summary score (54).

### Statistical Analysis

Statistical analyses were performed in Stata (Stata Statistical Software: Release 15. College Station, TX: StataCorp LLC) (55) using commands for complex survey designs when adopting logistic regression. The survey commands provide population-level estimates, by adjusting standard errors and incorporating stratification weights (28). Main exposures for this study were fruit and vegetable intake, combined and separately. Primary outcomes included the four domains of perceived stress (worries, tension, joy and demands). The association between the exposures and the odds of being in the highest quartile of each specific perceived stress domains was assessed using logistic regression. Statistical significance was set at *p* ≤ 0.05 (two-tailed) for all tests. Two models were considered: unadjusted and the multivariable adjusted for age, sex, BMI, energy intake, relationship status, physical activity levels, level of education, SEIFA, smoking status, self-reported history of CVD, and diabetes, as these covariates could affect both the exposure and outcomes of the study.

To further examine whether a healthier diet in general could confound the observed associations, we adjusted for diet quality (49). Income was used as a further adjustment for socio-economic status at the level of the individual. The life event scale was added to the multivariable adjusted model to adjust for the influence of major life events (e.g., death of a family member/friend) on the results. Finally, a physical health score was also added to the adjusted model to account for physical issues.

#### Additional Analyses

Generalized linear models adopting restricted cubic splines in R (56) were also used to examine the relationship between FV intake and each of the four perceived stress domains on a continuous scale.

Interaction terms between fruit and vegetable intake, separately, and age (<45 y, 45–65 y, ≥65 y), sex and physical activity were used to investigate potential effect modification domains where a significant overall relationship was observed.

## Results

### Baseline Characteristics

Baseline characteristics of all 8,640 participants is shown in Table 1. Participants' mean ± SD age was 47.8 ±15 years, with 50.1% being female. Median (IQR) scores for the perceived stress domains (maximum score of 20) including worries, tension, lack of joy and demands were 8 (6–10), 9 (7–12), 9 (7–11), and 10 (7–10), respectively. Participants who were excluded from the study presented similar characteristics (Supplementary Table 4).

**Table 1:** Clinical and demographic characteristics of all participants^a^.

**Characteristics**	**All**
**Sample size** (*n*)	**8,640**
**Age (years)**, mean ± SD	47.8 ± 15.0
**Sex (women)**, *n* (%)	4,747 (50.1)
**BMI (kg/m**^**2**^**)**, mean ± SD	26.6 ± 4.8
*Underweight, n* (%)	79 (1%)
*Normal weight, n* (%)	3,152 (39%)
*Overweight, n* (%)	3,483 (40%)
*Obese, n* (%)	1,926 (20%)
**Energy intake (kcal/d)**, mean ± SD	2,042 ± 697
**Physical activity (min/week)**, mean (IQR)	277 (258–295)
*Insufficient physical activity, n* (%)	4,142 (48%)
*Sufficient physical activity, n* (%)	4,498 (52%)
**Relationship status**, *n* (%)	
*Married*	6,268 (72.8)
*De facto*	414 (4.4)
*Separated*	217 (2.4)
*Divorced*	515 (5.1)
*Widowed*	528 (5.2)
*Single*	698 (10.0)
**SEIFA**	1,025 ± 87
**Level of education**, *n* (%)	
*Never to some high school*	3,445 (35.7)
*Completed university or equivalent*	5,195 (64.3)
**Smoking status**, ***n*** (%)	
*Current*	1,338 (16.3)
*Ex-smoker*	2,537 (25.9)
*Non-smoker*	4,765 (57.8)
**Prior CVD**, ***n*** (%)	685 (6.7)
**Diabetes status**, ***n*** (%)	
*Normal glucose levels*	6,387 (77.1)
*Impaired fasting glucose*	512 (5.8)
*Impaired glucose tolerance*	1,043 (10.3)
*Newly diagnosed diabetes mellitus*	350 (3.5)
*Known diabetes mellitus*	348 (3.3)
**Score for worries**, median (IQR)	8 (6–10)
**Score for tension**, median (IQR)	9 (7–12)
**Score for lack of joy**, median (IQR)	9 (7–11)
**Score for demands**, median (IQR)	10 (7–13)

a *Estimated using the survey command to apply the necessary weighting for selection bias. Worries, tension lack of joy and demands represent Q4. BMI, Body Mass Index; CVD, Cardiovascular Disease; SEIFA, Socio-Economical Index For Areas*.

### Associations of Fruit and Vegetable Intake With Perceived Stress Domains

When we examined the associations of the highest vs. lowest intakes of FV in those with higher perceived stress domains (top 25%), compared to participants with the lowest FV intake, those with the highest FV intake (Q4) had 32, 36, and 23% lower odds of worries, tension, and lack of joy, respectively (Table 2). There was, however, no association between FV intake and the demands domain of perceived stress.

**Table 2:** Odds ratios (OR) for worries, tension, lack of joy and demands by quartiles of FV intake.

	**Fruit and vegetable (FV) intake quartiles**			
	**Q1**	**Q2**	**Q3**	**Q4**
***FV intake***	*n = 2,161*	*n = 2,161*	*n = 2,158*	*n = 2,160*
Median (IQR) (g/day)	193 (155–221)	293 (269–318)	400 (371–436)	575 (518–676)
**Worries**, *n* (%)	860 (43%)	756 (38%)	641 (33%)	664 (32%)
Unadjusted	Ref.	0.80 (0.64, 1.01)	0.65 (0.54, 0.78)^#^	0.63 (0.52, 0.75)^#^
Multivariable adjusted	Ref.	0.82 (0.66, 1.02)	0.71 (0.58, 0.88)^#^	0.68 (0.55, 0.85)^#^
Multivariable adjusted plus income	Ref.	0.83 (0.67, 1.03)	0.72 (0.59, 0.89)^#^	0.69 (0.55, 0.86)^#^
Multivariable adjusted plus life events	Ref.	0.87 (0.67, 1.11)	0.74 (0.58, 0.94)^*^	0.73 (0.57, 0.93)^*^
Multivariable adjusted plus physical health score	Ref.	0.82 (0.66, 1.02)	0.72 (0.59, 0.89)^#^	0.70 (0.56, 0.89)#
**Tension**, *n* (%)	751 (37%)	643 (32%)	547 (30%)	542 (26%)
Unadjusted	Ref.	0.79 (0.64, 0.98)^*^	0.72 (0.57, 0.90)^#^	0.58 (0.49, 0.70) ^#^
Multivariable adjusted	Ref.	0.81 (0.65, 1.00)	0.78 (0.62, 0.98)^*^	0.64 (0.52, 0.79)^#^
Multivariable adjusted plus income	Ref.	0.81 (0.66, 1.00)	0.78 (0.62, 0.99)^*^	0.64 (0.52, 0.79)^#^
Multivariable adjusted plus life events	Ref.	0.82 (0.65, 1.03)	0.83 (0.64, 1.07)	0.67 (0.54, 0.84)^#^
Multivariable adjusted plus physical health score	Ref.	0.84 (0.69, 1.02)	0.79 (0.63, 0.99)^*^	0.66 (0.54, 0.82)^#^
**Lack of joy**, *n* (%)	744 (35%)	646 (32%)	561 (27%)	525 (27%)
Unadjusted	Ref.	0.86 (0.68, 1.08)	0.67 (0.54, 0.83)^#^	0.68 (0.57, 0.80)^#^
Multivariable adjusted	Ref.	0.89 (0.71, 1.13)	0.76 (0.60, 0.95)^*^	0.77 (0.64, 0.93)^#^
Multivariable adjusted plus income	Ref.	0.90 (0.72, 1.14)	0.76 (0.60, 0.96)^*^	0.78 (0.64, 0.94)^#^
Multivariable adjusted plus life events	Ref.	0.93 (0.74, 1.16)	0.79 (0.62, 1.00)^*^	0.81 (0.68, 0.97)^*^
Multivariable adjusted plus physical health score	Ref.	0.92 (0.74, 1.14)	0.77 (0.61, 0.97)^*^	0.78 (0.64, 0.96)^*^
**Demands**, *n* (%)	711 (37%)	671 (34%)	567 (31%)	565 (30%)
Unadjusted	Ref.	0.90 (0.71, 1.14)	0.79 (0.65, 0.95)^*^	0.74 (0.60, 0.91)^#^
Multivariable adjusted	Ref.	0.92 (0.68, 1.23)	0.88 (0.72, 1.08)	0.88 (0.69, 1.13)
Multivariable adjusted plus income	Ref.	0.89 (0.66, 1.22)	0.87 (0.71, 1.07)	0.87 (0.67, 1.12)
Multivariable adjusted plus life events	Ref.	0.93 (0.68, 1.28)	0.91 (0.73, 1.13)	0.94 (0.72, 1.22)
Multivariable adjusted plus physical health score	Ref.	0.94 (0.71, 1.24)	0.89 (0.73, 1.08)	0.89 (0.69, 1.15)

*Odds ratios (ORs) and 95% confidence intervals obtained using the survey command for logistic regression. Cut-points for worries, tension, lack of joy and demands were ≥9, ≥11, ≥11, ≥12 for men; and ≥10, ≥12, ≥12, ≥13 for women, respectively. Analyses were unadjusted and multivariable adjusted for age, sex, BMI [body mass index], energy intake, relationship status, physical activity, level of education, SEIFA [Socio-economical index for areas], smoking status, diabetes and prior cardiovascular disease).*

* *Indicates a statistically significant difference (p < 0.05) from Q1;*

# *indicates a statistically significant difference (p <0.013) from Q1 based on Bonferroni-corrections*.

The associations between fruit intake alone and each domain of perceived stress are shown in Table 3. Compared to participants with the lowest fruit intake, those with the highest fruit intake had 25, 27, and 16% lower odds for worries, tension, and lack of joy, respectively. No relationship was observed for the demands domain.

**Table 3:** Odds ratios (OR) for worries, tension, lack of joy and demands by quartiles of fruit intake.

	**Fruit intake quartiles**			
	**Q1**	**Q2**	**Q3**	**Q4**
***Fruit intake***	*n = 2,160*	*n = 2,160*	*n = 2,161*	*n = 2,159*
Median (IQR) (g/day)	62 (52–72)	119 (106–133)	225 (193–250)	372 (322–448)
**Worries**, ***n*** **(%)**	803 (40%)	711 (36%)	733 (36%)	674 (33%)
Unadjusted	Ref.	0.69 (0.58, 0.82)^#^	0.58 (0.44, 0.75)^#^	0.61 (0.52, 0.71)^#^
Multivariable adjusted	Ref.	0.80 (0.67, 0.96)^*^	0.74 (0.58, 0.95)^*^	0.75 (0.62, 0.89)^#^
Multivariable adjusted plus income	Ref.	0.80 (0.67, 0.95)^*^	0.75 (0.59, 0.95)^*^	0.75 (0.63, 0.90)^#^
Multivariable adjusted plus life events	Ref.	0.84 (0.67, 1.06)	0.77 (0.55, 1.09)	0.80 (0.65, 0.98)^*^
Multivariable adjusted plus physical health score	Ref.	0.82 (0.69, 0.97)^*^	0.75 (0.59, 0.95)^*^	0.77 (0.63, 0.94)^#^
**Tension**, ***n*** **(%)**	765 (37%)	604 (32%)	577 (29%)	537 (27%)
Unadjusted	Ref.	0.80 (0.66, 0.97)^*^	0.68 (0.57, 0.82)^#^	0.63 (0.54, 0.74)^#^
Multivariable adjusted	Ref.	0.85 (0.71, 1.02)	0.79 (0.65, 0.96)^*^	0.73 (0.62, 0.86)^#^
Multivariable adjusted plus income	Ref.	0.85 (0.71, 1.01)	0.79 (0.65, 0.96)^*^	0.73 (0.62, 0.86)^#^
Multivariable adjusted plus life events	Ref.	0.88 (0.69, 1.12)	0.84 (0.65, 1.07)	0.77 (0.63, 0.93)^#^
Multivariable adjusted plus physical health score	Ref.	0.85 (0.72, 1.00)	0.78 (0.64, 0.96)^*^	0.74 (0.62, 0.87)^#^
**Lack of joy**, ***n*** **(%)**	778 (37%)	590 (29%)	581 (27%)	527 (28%)
Unadjusted	Ref.	0.71 (0.62, 0.81)^#^	0.63 (0.51, 0.77)^#^	0.66 (0.56, 0.77)^#^
Multivariable adjusted	Ref.	0.84 (0.73, 0.96)^#^	0.80 (0.64, 1.01)	0.84 (0.71, 0.99)^*^
Multivariable adjusted plus income	Ref.	0.84 (0.73, 0.96)^#^	0.81 (0.65, 1.01)	0.84 (0.71, 1.00)
Multivariable adjusted plus life events	Ref.	0.88 (0.75, 1.03)	0.85 (0.68, 1.07)	0.89 (0.73, 1.08)
Multivariable adjusted plus physical health score	Ref.	0.85 (0.74, 0.96)^*^	0.79 (0.63, 0.99)^*^	0.86 (0.70, 1.04)
**Demands**, ***n*** **(%)**	715 (37%)	635 (34%)	601 (30%)	563 (31%)
Unadjusted	Ref.	0.86 (0.72, 1.03)	0.73 (0.58, 0.92)^#^	0.78 (0.65, 0.93)^#^
Multivariable adjusted	Ref.	0.93 (0.74, 1.16)	0.89 (0.67, 1.18)	0.97 (0.79, 1.18)
Multivariable adjusted plus income	Ref.	0.93 (0.73, 1.18)	0.87 (0.65, 1.17)	0.95 (0.77, 1.18)
Multivariable adjusted plus life events	Ref.	0.97 (0.74, 1.26)	0.92 (0.65, 1.29)	1.02 (0.81, 1.30)
Multivariable adjusted plus physical health score	Ref.	0.91 (0.72, 1.16)	0.88 (0.66, 1.17)	0.95 (0.77, 1.18)

*Odds ratios (ORs) and 95% confidence intervals obtained using the survey command for logistic regression. Cut-points for worries, tension, lack of joy, and demands were ≥9, ≥11, ≥11, ≥12 for men; and ≥10, ≥12, ≥12, ≥13 for women, respectively. Analyses were unadjusted and multivariable adjusted for age, sex, BMI [body mass index], energy intake, relationship status, physical activity, level of education, SEIFA [Socio-economical index for areas], smoking status, diabetes and prior cardiovascular disease).*

* *Indicates a statistically significant difference (p <0.05) from Q1;*

# *indicates a statistically significant difference (p <0.013) from Q1 based on Bonferroni-corrections*.

The associations between vegetable consumption alone and domains of perceived stress are shown in Table 4. Compared to individuals with lower intake, those with the highest vegetables intake (Q4) had a statistically significant 29, 28, and 33% lower odds for worries, tension, and lack of joy, respectively. No relationship was observed for the demand domain.

**Table 4:** Odds ratios (OR) for worries, tension, lack of joy and demands by quartiles of vegetable intake.

	**Vegetable intake quartiles**			
	**Q1**	**Q2**	**Q3**	**Q4**
***Vegetable intake***	*n = 2,160*	*n = 2,163*	*n = 2,157*	*n = 2,160*
Median (IQR) (g/day)	89 (65–105)	142 (130–153)	188 (175–202)	264 (238–308)
**Worries**, ***n*** **(%)**	888 (45%)	705 (36%)	669 (32%)	659 (33%)
Unadjusted	Ref.	0.84 (0.69, 1.01)	0.85 (0.66, 1.10)	0.73 (0.58, 0.94)^*^
Multivariable adjusted	Ref.	0.95 (0.82, 1.11)	0.88 (0.67, 1.14)	0.71 (0.56, 0.90)^#^
Multivariable adjusted plus income	Ref.	0.96 (0.84, 1.11)	0.88 (0.67, 1.15)	0.72 (0.58, 0.90)^#^
Multivariable adjusted plus life events	Ref.	0.95 (0.75, 1.20)	0.89 (0.65, 1.21)	0.70 (0.52, 0.95)^*^
Multivariable adjusted plus physical health score	Ref.	0.96 (0.83, 1.11)	0.88 (0.67, 1.14)	0.72 (0.58, 0.91)^#^
**Tension**, ***n*** **(%)**	706 (36%)	608 (32%)	608 (30%)	561 (28%)
Unadjusted	Ref.	0.84 (0.65, 1.08)	0.76 (0.56, 1.02)	0.71 (0.57, 0.87)^#^
Multivariable adjusted	Ref.	0.91 (0.70, 1.16)	0.78 (0.57, 1.08)	0.72 (0.57, 0.90)^#^
Multivariable adjusted plus income	Ref.	0.91 (0.71, 1.17)	0.78 (0.57, 1.08)	0.72 (0.57, 0.90)^#^
Multivariable adjusted plus life events	Ref.	0.92 (0.69, 1.22)	0.79 (0.55, 1.12)	0.72 (0.55, 0.94)^*^
Multivariable adjusted plus physical health score	Ref.	0.93 (0.73, 1.18)	0.80 (0.59, 1.08)	0.74 (0.59, 0.92)^#^
**Lack of joy**, ***n*** **(%)**	727 (36%)	587 (29%)	588 (29%)	574 (28%)
Unadjusted	Ref.	0.72 (0.59, 0.89)^#^	0.71 (0.51, 1.00)	0.69 (0.52, 0.90)^#^
Multivariable adjusted	Ref.	0.81 (0.65, 1.01)	0.72 (0.51, 1.02)	0.67 (0.51, 0.89)^#^
Multivariable adjusted plus income	Ref.	0.81 (0.65, 1.01)	0.72 (0.51, 1.02)	0.68 (0.52, 0.89)^#^
Multivariable adjusted plus life events	Ref.	0.82 (0.66, 1.02)	0.72 (0.50, 1.05)	0.67 (0.49, 0.93)^*^
Multivariable adjusted plus physical health score	Ref.	0.83 (0.69, 1.00)	0.74 (0.54, 1.02)	0.69 (0.52, 0.90)^#^
**Demands**, ***n*** **(%)**	687 (36%)	642 (35%)	620 (30%)	565 (31%)
Unadjusted	Ref.	0.93 (0.78, 1.11)	0.75 (0.57, 0.98)^*^	0.78 (0.63, 0.98)^*^
Multivariable adjusted	Ref.	1.07 (0.88, 1.30)	0.82 (0.60, 1.11)	0.84 (0.67, 1.05)
Multivariable adjusted plus income	Ref.	1.05 (0.86, 1.29)	0.81 (0.60, 1.10)	0.82 (0.67, 1.02)
Multivariable adjusted plus life events	Ref.	1.08 (0.87, 1.33)	0.82 (0.59, 1.14)	0.86 (0.68, 1.08)
Multivariable adjusted plus physical health score	Ref.	1.08 (0.89, 1.31)	0.84 (0.62, 1.14)	0.85 (0.68, 1.06)

*Odds ratios (ORs) and 95% confidence intervals obtained using the survey command for logistic regression. Cut-points for worries, tension, lack of joy and demands were ≥9, ≥11, ≥11, ≥12 for men; and ≥10, ≥12, ≥12, ≥13 for women, respectively. Analyses were unadjusted and multivariable adjusted for age, sex, BMI [body mass index], energy intake, relationship status, physical activity, level of education, SEIFA [Socio-economical index for areas], smoking status, diabetes and prior of cardiovascular disease).*

* *Indicates a statistically significant difference (p < 0.05) from Q1;*

# *indicates a statistically significant difference (p < 0.013) from Q1 based on Bonferroni-corrections*.

The addition of a marker of diet quality into the multivariable adjusted model only slightly affected our results; specifically, the association of higher intake of fruit, and FV combined, with lower lack of joy was attenuated (Q4 for fruit, OR [95%CI] 0.93 [0.77, 1.13]; for FV, 0.87 [0.70, 1.07]), perhaps because the diet quality index includes fruit and vegetable in its calculation. This relationship for vegetable intake remained similar to the primary analysis (OR [95% CI]: 0.72 [0.55, 0.95]). Similar relationships were observed for higher FV intake with lower worries (Q4 for FV, 0.69 [0.54, 0.89]; for fruit, 0.77 [0.64, 0.93]; for vegetable, 0.73 [0.58, 0.94]), compared to Q4. Likewise, the relationship for higher FV intake and lower odds for tension (Q4 for FV, 0.70 [0.56, 0.87]; fruit, 0.80 [0.65, 0.98]; vegetable, 0.77 [0.61, 0.98]), compared to lower intake (Q1) remained similar.

Adding income, life events and a physical health score as further covariates to the multivariable adjusted model did not change the associations of FV intake (Table 2) with the domains of perceived stress, as well as with vegetable intake (Table 4). The association of fruit intake with worries and tension remained similar after further adjustments for the aforementioned covariates. However, the association of fruit intake with lack of joy did not remain significant after further adjusting for income, life events and physical health score (Table 3).

Excluding participants with diabetes (*n* = 698, 6.8%) did not alter the results of the study. Among participants without diabetes (*n* = 7,942), those with higher consumption of FV had 36, 40, and 27% lower odds of worries (0.64 [0.50, 0.80]), tension (0.60 [0.49, 0.73]), and lack of joy (0.73 [0.60, 0.89]), respectively. This was similar for the associations of fruit intake with worries (0.71 [0.59, 0.84]), tension (0.68 [0.59, 0.81]), and lack of joy (0.80 [0.66, 0.97]), and for vegetables with worries (0.69 [0.56, 1.11]), tension (0.71 [0.57, 0.88]), and lack of joy (0.67 [0.51, 0.87]).

### Additional Analyses

In multivariable adjusted general linear models, FV intake was non-linearly associated with worries, tension and lack of joy (all *p* for non-linearity <0.01) and linearly associated with demands (*p* for non-linearity = 0.59). Supplementary Figure 2 demonstrates the relationship of FV intake (g/day) with (a) worries, (b) tension, (c) lack of joy, and (d) demands (scores ranging from 5 to 20, lowest to highest; all *p* < 0.01).

In the interaction testing, sex and age did not influence the relationship of fruit and vegetables with worries, tension and lack of joy (all p_interaction_ > 0.1). We observed a significant interaction between physical activity and intake of vegetables for tension (p_interaction_ = 0.002). In those with sufficient weekly amount of physical activity (≥150 min/week) there was 19% lower odds (0.81 [0.71, 0.93]) for higher tension, for each serve increase in vegetable intake, but this was not statistically significant (0.95 [0.85, 1.07]) in those with insufficient physical activity (<150 min/week).

All four perceived stress domains were significantly intercorrelated (worries vs. tension: r = 0.71, worries vs. joy: r = 0.53, worries vs. demands: r = 0.50, tension vs. joy: r = 0.63, tension vs. demands: r = 0.57: all *p* < 0.001), with the correlation between joy and demands being the weakest (r = 0.33).

## Discussion

We have previously reported an inverse association between FV intake, combined and separately, with ~10% lower levels of perceived stress (8). We now extend our work and have examined the relationship of FV intake with four perceived stress domains (worries, tension, lack of joy and demands) for the first time in adults across all ages. When considering the four domains of perceived stress, we demonstrate that higher intakes of FV, combined and separately, were associated with lower odds for worries, tension, and lack of joy, independent of other lifestyle and socioeconomic factors. No relationship was observed for the environmental stressor “demands” domain.

It is noteworthy that fruit and vegetable data in this study was collected in 1999–2000. In 2017, according to the Australian Bureau of Statistics (57), the average fruit and vegetables consumption of adults (≥18 years) was approximately 1.7 serves of fruit (~250 g/d) and 2.4 serves of vegetables (~180 g/d) per day, respectively. Such findings are comparable to the average fruit and vegetable intake of 200 and 170 g/d, respectively, in the AusDiab cohort in 1999–2000. Although there was a slight increase in FV intake in the last 20 years, consumption of FV remains similar and below the recommendations.

A positive relationship of FV intake with lower odds of worries was seen in our study. This concurs with a previous study including ~8,500 year-5 Canadian students, where an inverse association between Diet Quality Index-International ([DQI-I]—consisting of four components: (1) variety [including FV groups], (2) adequacy [including FV], (3) moderation [i.e., sodium, total and saturated fats], and 4) balance [macronutrients and fatty acids ration]) (58) and feelings of worry (OR [95% CI]: 0.90 (0.85–0.97) was observed (59).

In our study, a higher intake of FV was associated with lower odds for tension. In support of our results, a previous study with 138 healthy men and women (60) observed lower scores of tension in those with a vegetarian diet, compared to omnivorous diets. Similarly, both a 14-week DASH (Dietary Approaches to Stop Hypertension) diet—including recommendation of ≥4 serves of fruit and ≥4 serves of vegetables, and a standard healthy diet—with recommendations of FV being ≥2 serves of fruit and 2–3 serves of vegetables, led to improvements in overall mood and other mood-related domains, including tension, in post-menopausal healthy women (61). All of the aforementioned diets are considered healthy diets and contain high amounts of FV, suggesting a beneficial role of FV-rich diets for tension.

Similar to our results on the positive relationship between greater intake of FV and joy, after examining the food diaries of more than 12,000 adults, a national Australian survey reported that consumption of FV predicted improvements in life satisfaction, happiness and well-being (62). Consuming a range of FV with different colors has also been positively linked to improved mood and increased creativity, curiosity, and conduciveness to happiness in young adults (63).

Bioactive compounds found in a healthy diet, particularly in a FV-rich diet, such as vitamins (i.e., B and C vitamins), minerals (i.e., zinc, magnesium) and fiber (i.e., resistant starch), have been linked to health and well-being (21). These constituents appear to play a protective role on mental health conditions (11), modulating some of the neurotransmitters in the brain, which regulate mood (21) and could potentially increase joy and happiness (64). Some neurochemicals (e.g., endorphin, dopamine, serotonin, norepinephrine, and melatonin) have been shown to cause feelings of happiness (22). Similarly, polyphenols found in FV have been positively associated with mental well-being, potentially due to their anti-inflammatory, neuroprotective, and prebiotic properties (65). Bioactive compounds in FV seem to protect against oxidative stress (23), inflammation (24) and brain plasticity (66), which are impaired in those with mental health issues (67). Dysbiosis of the gut microbiota is another potential pathway, with evidence showing the involvement of the microbiome in the modulation of stress response (11) and diet being an important modulator of gut health (68).

In the present study, consumption of FV was not associated with “demands.” We also observed that all four perceived stress domains were intercorrelated, with the “demands” domain showing the lowest correlation, as previously reported (25). This could be due to the nature of the “demands” domain, as demands is considered an external stressor, rather than a stress reaction (25). Also, feelings of demands have been reported to be stronger in middle-aged people, due to high career and social strains, in addition to caregiving responsibilities (69). By including people of all ages in the current study, potential demands perceived by participants may not have been as pronounced.

When we examined the relationship of FV intake as a continuous variable (g/day) with the scores of all four perceived stress domains, greater FV intake was significantly non-linearly associated with less worries, tension and lack of joy, and linearly associated with lower demands. Intake of FV >400 g/day appears to be more beneficial. A FV intake of approximately ≥400 g/day (median of Q3), was associated with lower odds of worries, tension and lack of joy. Such findings are supported by current recommendations by the World Health Organisation to consume a minimum of 400 g/day of FV for the prevention of non-communicable health conditions (70). When examining the relationship of fruit and vegetable separately, with perceived stress domains, we observed that consumption of ≥225 g/day (median in Q3) of fruit (≥1.5-serves per day), and at least 260 g/day of vegetables (at least 3.5 serves of vegetables) would provide the greatest benefits for perceived stress domains. Such findings also compliment Australian dietary guidelines (37) that recommend consuming 2 × 150 g serves of fruit and 5–6 × 75 g serves of vegetables each day as part of a healthy diet.

Another interesting finding of this study from the interaction analysis was that sex and age did not influence the relationship of FV with worries, tension and lack of joy. However, a significant interaction between physical activity and vegetable consumption for tension was observed. Upon further investigation, those with sufficient weekly amount of physical activity (≥150 min/week) had 19% lower odds for higher tension, for each serve increase in vegetable intake. Although the relationship between higher consumption of vegetable intake and lower tension (OR [95%CI]: 0.62 [0.41–0.93]) was significant in those with sufficient physical activity (≥150 min/week), despite a similar point estimate, this relationship was attenuated (0.83 [0.61–1.13]) in those with insufficient physical activity (0–150 min/week). Such findings highlight the importance of a high vegetable intake in combination with regular physical activity to minimize feelings of tension; findings also promoted previously (71). The importance of exercise for mental health disorders is well-established and likely to work in synergy with FV nutrients (71) to reduce tension. Although the mechanisms underlying the beneficial effects of exercise on well-being are not fully understood, some pathways have been suggested (72). Physical activity may improve psychological well-being by promoting self-efficacy (73). Physical activity has been associated with positive changes to insulin resistance, inflammation, and oxidative stress, which could potentially explain the beneficial effects of physical activity on cognitive function and brain-related diseases (74, 75). Evidence suggests that exercise (76), and a healthy diet (77) have a beneficial impact on gut microbiota diversity (76), which has been linked to better brain processes and behavior (78, 79). Current recommendations to increase FV intake for improved physical health may have positive benefits on mental health. Perhaps, a combination of the aforementioned lifestyle factors may work synergistically to further contribute toward positive mental health outcomes. For example, it is likely that the benefit of a healthy diet on perceived stress, specifically FV intake, is likely to be most beneficial when combined with regular exercise.

Overall, our results suggest a beneficial link between greater FV intake and lower worries, tension and lack of joy. However, the relationship between FV intake and stress is likely bidirectional, with an alternative interpretation of the associations being possible. Evidence shows that stress and mental health disorders can also lead to poor dietary habits, such as lower intake of FV and higher intake of energy-dense foods (13, 80).

### Limitations and Strengths of the Study

Limitations of this work include the cross-sectional nature of the study; therefore, we cannot infer causality. Despite AusDiab being a national survey including participants from 42 randomly selected census collector districts across all states and territories in Australia, designed to reflect the Australian general population, we cannot rule out the risk of selection bias. On the basis of the analysis, it's difficult to differentiate between a FV-rich diet leading to lower stress levels and improved well-being or vice versa. Based on previous evidence, it is likely that both co-exist. The mean BMI indicated our participants were slightly overweight (26.6 ± 4.8 kg/m^2^). Overeating and increased intake of high-dense energy and fat foods may exacerbate stress levels (19, 20). Therefore, caution should be exercised when generalizing these results to populations with substantially different BMI (e.g., underweight, severely obese). Approximately 7% of the participants had diabetes in 2000, which may have influenced the results of our study. As such, diabetes status was added to the multivariable adjusted model as a potential confounding factor to minimize potential bias. Although the findings from our study rely on a valid tool for assessing perceived stress domains in healthy populations (such us the participants from the AusDiab cohort), it is possible that these results may differ between cohorts due to differences in demographics.

Strengths of this study include the investigations of the relationship between FV intake and four domains of perceived stress, for the first time. Additionally, the sample size was large, national, population-based and included adults across the lifespan. Although most information was self-reported, both perceived stress and dietary intake were assessed using validated questionnaires.

Future studies could aim to further understand the potential mechanisms for the negative association between FV intake and perceived stress domains. There is also need for longitudinal studies to investigate the impact of FV on perceived stress domains, and vice-versa. The relationship between diet and stress, and stress-related mental health issues is likely to be bidirectional, and understanding its associations may have critical implications in alleviating stress levels and improving mental well-being (81). Clinical trials exploring the effects of FV intake on perceived stress domains would enhance understanding on the impact of FV intake on perceived stress domains, and vice-versa.

## Conclusion

Our data suggest that higher intakes of FV are inversely associated with overall stress perceived by adults, and specific stress domains, such as worries, tension and lack of joy. However, there is need for further research to examine the effects of overall and specific types of FV intake in a clinical trial setting.

## Data Availability Statement

The data presented in this article are not readily available because and belong to the Baker Heart and Diabetes Institute. Restrictions apply to the availability of these data, which were used under license for the current study. Raw data are not publicly available. However, data described in the manuscript, codebook, and analytic code will be made available upon request and approval of the AusDiab Steering Committee. Requests to access the datasets should be directed to jonathan.shaw@baker.edu.au.

## Author Contributions

SR-B, MS, and JL designed this cross-sectional study. DM, JS, and RD provided data collected in the original cohort study. SR-B and MS analyzed the data. SR-B drafted and had primary responsibility for final content of the manuscript. SR-B, MS, LB, NB, CB, RW, JD, CH, DM, JS, RD, JH, and JL interpreted the results, had significant input, reviewed, and approved the final manuscript. All authors contributed to the article and approved the submitted version.

## Funding

The salary of MS is supported by a Royal Perth Hospital Research Foundation Career Advancement Fellowship (ID: CAF 130/2020) and an Emerging Leader Fellowship from the Western Australian Future Health Research and Innovation Fund. The salary of LB was supported by a National Health and Medical Research Council (NHMRC) of Australia Emerging Leadership Investigator Grant (ID: 1172987) and a National Heart Foundation of Australia Post-Doctoral Research Fellowship (ID: 102498). The salary of CB is supported by a Royal Perth Hospital Research Foundation ‘Lawrie Beilin' Career Advancement Fellowship (ID: CAF 127/2020). The salary of JH was supported by a National Health and Medical Research Council of Australia Senior Research Fellowship (ID: 1116973). The salary of JS was supported by a National Health and Medical Research Council Investigator Grant (ID: 1173952). The salary of JL was supported by a National Heart Foundation of Australia Future Leader Fellowship (ID: 102817). None of the funding agencies had any role in the conduct of the study; collection, management, analysis, or interpretation of the data; or preparation, review, or approval of the manuscript.

## Conflict of Interest

CB and JH report grants from FruitWest, and from Department of Agriculture and Food WA, outside the submitted work. DM and JS report grants from Abbott Australasia Pty Ltd., Alphapharm Pty Ltd., AstraZeneca, Bristol-Myers Squibb, Eli Lilly Australia, GlaxoSmithKline, Janssen-Cilag, Merck Sharp and Dohme, Novartis Pharmaceuticals, Novo Nordisk Pharmaceuticals, Roche Diagnostics Australia, Sanofi Aventis and Sanofi-synthelabo, during the conduct of the study. RD received a Primary Growth Partnership grant via the Ministry of Primary Industries in New Zealand with Fonterra Co-operative Group Ltd., outside the submitted work. The remaining authors declare that the research was conducted in the absence of any commercial or financial relationships that could be construed as a potential conflict of interest. The reviewer LY declared a shared affiliation with the author RD to the handling editor at the time of review.

## Publisher's Note

All claims expressed in this article are solely those of the authors and do not necessarily represent those of their affiliated organizations, or those of the publisher, the editors and the reviewers. Any product that may be evaluated in this article, or claim that may be made by its manufacturer, is not guaranteed or endorsed by the publisher.
