# Supplementary material for: Higher Consumption of Fruit and Vegetables Is Associated With Lower Worries, Tension and Lack of Joy Across the Lifespan
Source: Front Nutr. 2022 May 2;9:837066. doi: 10.3389/fnut.2022.837066 (PMC9108498; doi:10.3389/fnut.2022.837066)
Supplement: Supplementary file 1 [file Data_Sheet_1.docx]

**Higher consumption of** **fruit and vegetables is associated with lower worries, tension and lack of joy across the lifespan**

**SUPPLEMENTARY MATERIAL**

**
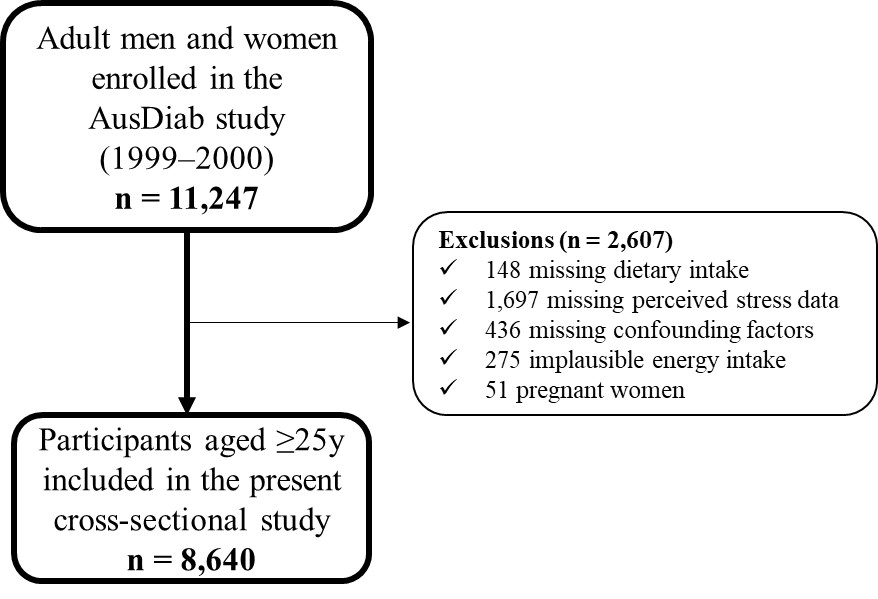
**

**Supplementary Figure 1.** Cross-sectional study flow diagram

**
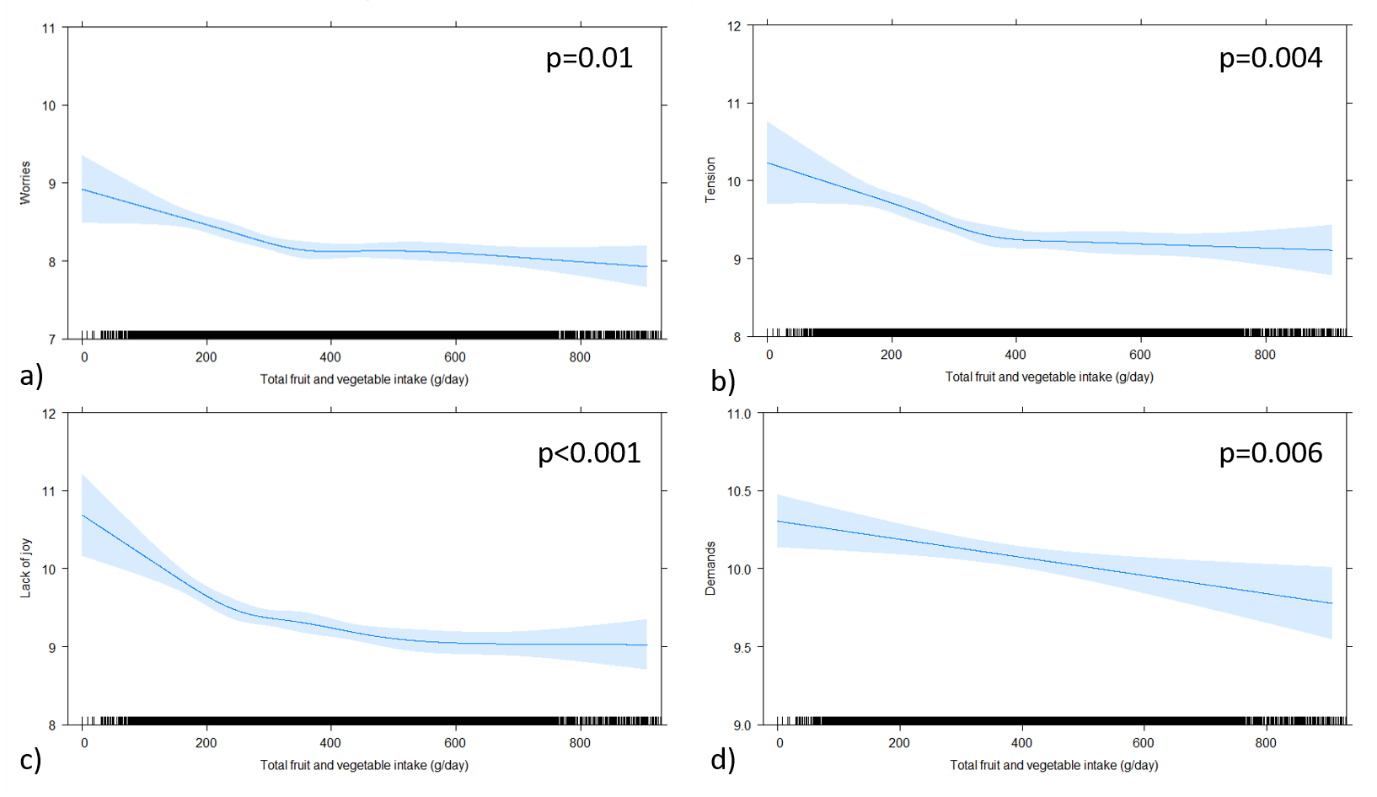
Supplementary Figure 2.** Illustration of the multivariable-adjusted relationship of fruit and vegetable intake (g/day) with a) Worries; b) Tension; c) Lack of joy; and d) Demands (n=8,640) obtained by general linear models with the exposure included as a restricted cubic spline. Shading area along the curve represents 95% confidence intervals. The rug plot along the bottom of each graph depicts each observation. All analyses were adjusted for age, sex, BMI (body mass index), energy intake, relationship status, physical activity, level of education, SEIFA (Socio-economical index for areas), smoking status, diabetes and prior cardiovascular disease. Perceived stress domains are given as a score, ranging from 5-20 (lowest to highest). P-values for the test of non-linearity were obtained using likelihood ratio tests to compare appropriate nested models

**Supplementary Table 1.** Specific items from the PSQ included in the four domains of perceived stress

| **Domains of perceived stress**  **(worries, tension, joy, and demands [stressor])*** | **30-item PSQ**  **(item numbers)**** |
| --- | --- |
| **“Worries”** |  |
| ✔ You fear you may not manage to attain your goals | Q.9 |
| ✔ You feel frustrated | Q.12 |
| ✔ Your problems seem to be piling up | Q.15 |
| ✔ You have many worries | Q.18 |
| ✔ You are afraid for the future | Q.22 |
| **x** You feel lonely or isolated | Q.5 |
| **x** You find yourself in situations of conflict | Q.6 |
| **x** You are under pressure from other people | Q.19 |
| **x** You feel discouraged | Q.20 |
| **x** You feel criticized or judged | Q.24 |
| **x** You feel you’re doing things because you have to not because you want to | Q.23 |
| **x** You feel loaded down with responsibility | Q.28 |
| **x** You have too many decisions to make | Q.11 |
| **“Tension”** |  |
| ✔ You feel rested (+) | Q.1 |
| ✔ You feel calm (+) | Q.10 |
| ✔ You feel tense | Q.14 |
| ✔ You feel mentally exhausted | Q.26 |
| ✔ You have trouble relaxing | Q.27 |
| **x** You are irritable or grouchy | Q.3 |
| **x** You feel tired | Q.8 |
| **“Joy”** |  |
| ✔ You feel you’re doing things you really like (+) | Q.7 |
| ✔ You are full of energy (+) | Q13 |
| ✔ You feel safe and protected (+) | Q.17 |
| ✔ You enjoy yourself (+) | Q.21 |
| ✔ You are light-hearted (+) | Q.25 |
| **“Demands”** |  |
| ✔ You feel that too many demands are being made on you | Q.2 |
| ✔ You have too many things to do | Q.4 |
| ✔ You feel you’re in a hurry | Q.16 |
| ✔ You have enough time for yourself (+) | Q.29 |
| ✔ You feel under pressure from deadlines | Q.30 |

✔Items from the original perceived stress questionnaire (PSQ) included in the domains.

**x** Items from the original PSQ not included in the perceived stress domains.

(+) Positive (reverse scored) questions.

*Domains of stress created by Fliege and colleagues (Fliege et al., 2005) – items were selected from the original 30-item PSQ and originated the four factors of perceived stress, based on exploratory factor analysis with promax-rotation (Fliege et al., 2005).

**30-item PSQ (Levenstein et al., 1993).

**Supplementary Table 2.** Cut-offs for quartiles of each perceived stress domains by sex

| **Domain’s quartiles** | **Scores for Men (n=3,893)** | **Scores for Women (n=4,747)** |
| --- | --- | --- |
| **Worries** |  |  |
| **Q1** | 0-5 | 0-5 |
| **Q2** | 6-7 | 6-7 |
| **Q3** | 8 | 8-9 |
| **Q4** | ≥9 | ≥10 |
| **Tension** |  |  |
| **Q1** | 0-6 | 0-6 |
| **Q2** | 7 | 7-8 |
| **Q3** | 8-10 | 9-11 |
| **Q4** | ≥11 | ≥12 |
| **Lack of joy** |  |  |
| **Q1** | 0-6 | 0-6 |
| **Q2** | 7-8 | 7-8 |
| **Q3** | 9-10 | 9-11 |
| **Q4** | ≥11 | ≥12 |
| **Demands** |  |  |
| **Q1** | 0-6 | 0-6 |
| **Q2** | 7-8 | 7-9 |
| **Q3** | 9-11 | 10-12 |
| **Q4** | ≥12 | ≥13 |

Q4 represents higher worries, tension, lack of joy, and demands

**Supplementary table 3.** Individual fruit and vegetables used to assess total intake of fruit and vegetables

| **Fruit** | **Vegetables** | | |
| --- | --- | --- | --- |
| 1. Oranges and other citrus fruits | 1. Potatoes cooked without fat | **13**. Silver beet or spinach |  |
| 1. Apples | 1. Tomato | **14**. Peas |  |
| 1. Pears | 1. Tomato sauce/paste or dried tomatoes | **15**. Green beans |  |
| 1. Bananas | 1. Peppers (capsicums) | **16**. Bean sprots or alfalfa sprouts |  |
| 1. Watermelon, rockmelon, honeydew | 1. Lettuce and other salad greens | **17.** Baked beans |  |
| 1. Pineapple | 1. Cucumber | **18**. Soybeans, soybean curd or tofu |  |
| 1. Strawberry | 1. Celery | **19**. Other beans (i.e. chick peas and lentils) |  |
| 1. Apricots | 1. Beetroot | **20**. Pumpkin |  |
| 1. Peaches and nectarines | 1. Carrots | **21**. Onion or leek |  |
| 1. Mango and pawpaw | 1. Cabbage and Brussels sprouts | **22**. Garlic |  |
| 1. Avocado | 1. Cauliflower | **23**. Mushrooms |  |
|  | 1. Broccoli | **24**. Zucchini |  |

Total intake of fruit and vegetables (in grams per day) were calculated by summing each of the fruit (11 items) and vegetables (24 items) listed on the table, based on the FFQ from the Cancer Council of Victoria (Ireland et al., 1994)

**Supplementary Table 4.** Clinical and demographic characteristics of excluded participants^1^

| **Characteristics** | **Excluded** | **Sample size (n)** |
| --- | --- | --- |
| **Age (years), mean ± SD** | 49.7 ± 17.0 | 2,607 |
| **Sex (women), n (%)** | 2,451 (53.9) | 2,607 |
| **BMI (kg/m^2^), mean ± SD** | 26.8 ± 5.2 | 2,427 |
| **Energy intake (kcal/d), mean ± SD** | 2,156 ± 1463 | 2,607 |
| **Physical activity, min/week** | 262 ± 340 | 2,607 |
| **Relationship status, n (%)** |  |  |
| *Married* | 1,771 (66) | 2,600 |
| *De facto* | 116 (5) |  |
| *Separated* | 76 (3) |  |
| *Divorced* | 165 (6) |  |
| *Widowed* | 251 (9) |  |
| *Single* | 222 (11) |  |
| **SEIFA** | 1,012 ± 85 | 2,515 |
| **Level of education, n (%)** |  |  |
| *Never to some high school* | 1,158 (43) | 2,546 |
| *Completed university or equivalent* | 1,388 (57) |  |
| **Smoking status, n (%)** |  | 2,395 |
| *Current* | 406 (14) |  |
| *Ex-smoker* | 681 (24) |  |
| *Non-smoker* | 1,308 (52) |  |
| **Prior CVD, n (%)** | 254 (10) | 2,465 |
| **Prevalence of diabetes, n (%)** |  | 2,607 |
| *Known diabetes* | 127 (4) |  |
| *Newly diagnosed diabetes* | 169 (8) |  |
| *Impaired fasting glucose* | 138 (5) |  |
| *Impaired glucose tolerance* | 335 (11) |  |
| *Normal glucose levels* | 1,719 (68) |  |
| **Score for worries,** **median (IQR)** | 8 (6-10) | 1,176 |
| **Score for tension,** **median (IQR)** | 9 (7-12) | 1,215 |
| **Score for lack of joy,** **median (IQR)** | 9 (7-12) | 1,199 |
| **Score for demands,** **median (IQR)** | 10 (7-13) | 1,254 |

^1^Estimated using the survey command to apply the necessary weighting for selection bias. Worries, tension lack of joy and demands represent Q4. BMI, Body Mass Index; CVD, Cardiovascular Disease; SEIFA, Socio-Economical Index For Areas.

**References**

Fliege, H., Rose, M., Arck, P., Walter, O.B., Kocalevent, R.-D., Weber, C., et al. (2005). The Perceived Stress Questionnaire (PSQ) reconsidered: validation and reference values from different clinical and healthy adult samples. *Psychosomatic medicine* 67(1)**,** 78-88.

Ireland, P., Jolley, D., Giles, G., O’Dea, K., Powles, J., Rutishauser, I., et al. (1994). Development of the Melbourne FFQ: a food frequency questionnaire for use in an Australian prospective study involving an ethnically diverse cohort. *Asia Pac J Clin Nutr* 3(1)**,** 19-31.

Levenstein, S., Prantera, C., Varvo, V., Scribano, M.L., Berto, E., Luzi, C., et al. (1993). Development of the Perceived Stress Questionnaire: a new tool for psychosomatic research. *Journal of psychosomatic research* 37(1)**,** 19-32.
